# Efficacy and safety of vibegron for the treatment of irritable bowel syndrome in women: Results of a randomized, double‐blind, placebo‐controlled phase 2 trial

**DOI:** 10.1111/nmo.14448

**Published:** 2022-08-16

**Authors:** Brian E. Lacy, Jennifer King, Denise Shortino, Chris Schaumburg, Cornelia Haag‐Molkenteller, William D. Chey

**Affiliations:** ^1^ Gastroenterology and Hepatology Mayo Clinic Jacksonville Florida USA; ^2^ Urovant Sciences Irvine California USA; ^3^ Division of Gastroenterology University of Michigan Health System Ann Arbor Michigan USA

**Keywords:** abdominal pain, constipation, diarrhea, functional colonic diseases, gastrointestinal diseases, irritable bowel syndrome

## Abstract

**Background:**

Preclinical and clinical studies suggest that β_3_‐adrenergic receptor activation may be a novel target for treating abdominal pain and gastrointestinal motility dysfunction in patients with irritable bowel syndrome (IBS). This proof‐of‐concept study evaluated the efficacy and safety of the β_3_‐adrenergic agonist vibegron in treating IBS‐related pain.

**Methods:**

Adult women with predominant‐diarrhea IBS (IBS‐D) or with mixed diarrhea/constipation (IBS‐M), diagnosed using Rome IV criteria, were randomized 1:1 to receive once‐daily vibegron 75 mg or placebo for 12 weeks. The primary endpoint was the percentage of patients with IBS‐D considered abdominal pain intensity (API) weekly responders, defined as ≥30% reduction from baseline at week 12 in mean weekly worst abdominal pain over 24 hours using the API score. Patients completed a pain diary at baseline and at weeks 2, 4, 8, and 12. Safety was assessed by adverse events (AEs) in the overall IBS population.

**Key Results:**

Of the 222 patients with IBS randomized (vibegron, *N* = 111; placebo, *N* = 111), 85% completed the trial. There was no significant difference in the percentage of patients with IBS‐D (vibegron, *N* = 66; placebo, *N* = 63) considered API weekly responders with vibegron vs. placebo (*p* = 0.8222) after 12 weeks. The incidence of AEs was comparable between treatment groups (33.3% each), with equal rates of worsening IBS symptoms (2.7% each).

**Conclusions and Inferences:**

In women with IBS‐D, vibegron was not associated with significant improvement in the percentage of API weekly responders. Vibegron was generally safe and well tolerated and, in particular, did not worsen IBS symptoms vs. placebo.

## Graphical abstract

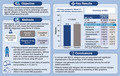

**Key Points**

- In this phase 2 proof‐of‐concept study, the β_3_‐adrenergic receptor agonist vibegron was not associated with significant improvement in the percentage of abdominal pain intensity weekly responders in adult women with irritable bowel syndrome (IBS) and predominant diarrhea.
- Vibegron was generally safe and well tolerated and was not associated with worsening of IBS symptoms compared with placebo.

## 1 INTRODUCTION

Irritable bowel syndrome (IBS) is a chronic disorder of gut‐brain interaction (DGBI) with an approximate worldwide prevalence of 4.1% in adults using the Rome IV criteria.^1^, ^2^ IBS, the most commonly diagnosed DGBI, is a symptom complex characterized by altered bowel habits with recurrent abdominal pain, as well as bloating, distention, and urgency.^3^, ^4^, ^5^ Symptoms of IBS are more commonly reported in women than in men.^1^ By definition, there are no gross biochemical, radiologic, or endoscopic findings present to account for IBS symptoms, and the pathophysiology is complex and multifactorial.^6^, ^7^ Therefore, the Rome IV criteria were developed for standardizing diagnostic criteria of IBS, which is subtyped based on the predominant stool pattern including diarrhea (IBS‐D), constipation (IBS‐C), or mixed episodes of diarrhea and constipation (IBS‐M).^7^

Similar to the clinical presentation, the pathogenesis of IBS is heterogeneous. Certain environmental and host factors—such as stress, antibiotics, enteric infections, food intolerances, and altered gut‐brain interactions—may alter gastrointestinal function and sensation, enabling the development of IBS symptoms.^3^ Still, the underlying pathophysiologic mechanisms associated with IBS are incompletely understood, making treatment of this condition challenging. Current dietary recommendations for the management of IBS from the American College of Gastroenterology (ACG) include reducing intake of fermentable oligosaccharides, disaccharides, monosaccharides, and polyols (FODMAPs) and incorporating soluble fiber.^6^ The ACG also recommends using pharmacologic therapies including chloride channel and guanylate cyclase activators to treat IBS‐C, as well as rifaximin (a non‐absorbed antibiotic), alosetron (a 5‐HT_3_ antagonist), and eluxadoline (a mixed μ‐ and κ‐opioid receptor agonist/δ‐opioid receptor antagonist) for managing severe symptoms in patients with IBS‐D.^6^ Unfortunately, despite these treatment options, many patients with IBS experience persistent symptoms.

β_3_‐adrenergic receptors are expressed and distributed throughout the gastrointestinal tract, including nonvascular smooth muscle and enteric neurons of the colon, both of which play a key role in gastrointestinal motility.^8^, ^9^, ^10^ Therefore, β_3_‐adrenergic receptor activation has been suggested as a potential novel target for treating pain and modulating gastrointestinal motility in patients with IBS.^11^, ^12^ Evidence for the therapeutic potential of β_3_‐adrenergic receptor activation in the treatment of IBS has been shown in preclinical studies, including ex vivo data showing β_3_‐adrenergic receptor agonist inhibition of cholinergic contractions in isolated human colon, which was fully reversed in the presence of a β_3_‐adrenergic receptor antagonist.^10^ A pilot clinical trial demonstrated improved IBS‐related pain among women with IBS receiving a β_3_‐adrenergic receptor agonist.^12^

Vibegron is a selective agonist of β_3_‐adrenergic receptors and was recently approved in the United States and Japan for the treatment of overactive bladder (OAB) in adults.^13^, ^14^ Vibegron showed efficacy and safety for the treatment of OAB in phase 3 trials.^15^, ^16^ Given the therapeutic potential of β_3_‐adrenergic receptor agonists in the treatment of IBS symptoms based on the nonclinical and clinical pilot data and the demonstrated safety of vibegron for OAB, we evaluated our hypothesis that women with IBS‐D or IBS‐M, treated with the same dose of vibegron as in the OAB trials, would have a greater improvement in IBS‐related pain than women treated with placebo in this phase 2 proof‐of‐concept study.

## 2 METHODS

### 2.1 Study design and participants

This phase 2, randomized, double‐blind, placebo‐controlled, parallel‐group, multicenter clinical trial (ClinicalTrials.gov identifier, NCT03806127) was conducted as a proof‐of‐concept study to evaluate the efficacy and safety of vibegron in adult women with IBS. The study was conducted in compliance with Good Clinical Practice, and an institutional review board at each participating site approved the study. All patients who participated in the study provided written informed consent.

Female patients who were 18–70 years of age were enrolled if they had an established history of IBS‐D or IBS‐M according to the Rome IV criteria, including recurrent abdominal pain, on average ≥1 day per week in the last 3 months, with symptom onset ≥6 months before diagnosis.^4^ Criteria for IBS‐D were determined by the predominant stool pattern present including loose, mushy, or watery stools (Bristol Type 6 or 7) for >25% of bowel movements and hard or lumpy stools (Bristol Type 1 or 2) for <25% of bowel movements. Up to 50% of patients could have IBS‐M with criteria including hard or lumpy stools (Bristol Type 1 or 2) for >25% of bowel movements and loose, mushy, or watery stools (Bristol Type 6 or 7) for >25% of bowel movements. Exclusion criteria included diagnosis of IBS‐C or IBS with unknown subtype per Rome IV criteria; history of chronic idiopathic constipation or functional constipation; structural abnormality of the gastrointestinal tract or a disease (e.g., known small intestine bacterial overgrowth) or condition that can affect gastrointestinal motility; history of a gastrointestinal motility disorder other than IBS (e.g., gastroparesis, intestinal pseudo‐obstruction, achalasia, Parkinson disease, multiple sclerosis, spinal cord injury); prior history of a gastrointestinal malignancy, inflammatory bowel disease, or celiac disease; planned gastrointestinal or abdominal surgery within the next 6 months; coexisting gastroesophageal reflux disease or functional dyspepsia with symptoms predominant to IBS symptoms; and symptoms or diagnosis of a medical condition other than IBS that could account for abdominal pain (e.g., interstitial cystitis, fibromyalgia currently being treated with pregabalin or gabapentin, and endometriosis with uncontrolled abdominal pain). No exclusions were made due to hemorrhoids. There were no dietary restrictions during the study period. Patients who had received any investigational agent within 28 days of the start of the study were excluded, as were women who were pregnant, nursing, or planning a pregnancy. Patients were permitted to remain on certain medications, including antidepressants, provided they were on a stable dose. Rescue medications for pain (equivalent of ibuprofen 400 mg twice daily or less; acetaminophen 500 mg three times daily or less; aspirin ≤325 mg/day), constipation (polyethylene glycol; bisacodyl ≤5 mg weekly), and diarrhea (loperamide ≤4 mg four times daily) were permitted at day 1 or after.

The trial consisted of a 1‐ to 5‐week screening period; a 2‐week single‐blind run‐in period; a 12‐week, randomized, double‐blind treatment period; and a 2‐week safety follow‐up period. Patients meeting the appropriate inclusion criteria were randomly assigned in a 1:1 ratio to receive once‐daily vibegron 75 mg or placebo for 12 weeks. Randomization was stratified by baseline abdominal pain intensity (API) score (<6 vs. ≥6 on a 0‐ to 10‐point numeric rating scale [NRS]) and IBS subtype (IBS‐D vs. IBS‐M). Patients completed an event‐driven bowel movement diary and a daily pain diary to assess and rate worst abdominal pain over 24 hours using the 0‐ to 10‐point NRS at baseline and at weeks 2, 4, 8, and 12. Patients also completed the Global Improvement Scale (GIS; 7‐point Likert scale, from substantially worse to substantially improved)^17^, ^18^ at weeks 2, 4, 8, and 12 to assess whether their IBS symptoms were either moderately or significantly relieved.

This study was conducted at the start of the COVID‐19 pandemic; most patients (87.7%) did not have any study visits affected by COVID‐19.

### 2.2 Assessments

#### 2.2.1 Efficacy endpoints

The primary objective of this phase 2 proof‐of‐concept study was to estimate the treatment effect of vibegron vs. placebo in improving IBS‐related abdominal pain in women with IBS‐D. The primary efficacy endpoint was the percentage of patients with IBS‐D who were API weekly responders over 12 weeks, defined as a patient who experienced a ≥30% reduction from baseline at week 12 in the weekly average of “worst abdominal pain in the past 24 hours” on the API score. A patient was considered an API weekly responder over weeks 1–12 if they met the API weekly responder criteria for ≥50% of the weeks assessed (i.e., ≥6 weeks).

Secondary efficacy endpoints included the percentage of patients with IBS‐D or IBS‐M who were considered GIS responders, defined as patients who reported that their IBS symptoms were either moderately or significantly relieved, and the percentage of patients with IBS‐D considered API responders over 12 weeks, defined as ≥40% and ≥50% reduction from baseline at week 12 in the weekly average of “worst abdominal pain in the past 24 hours” on the API score.

Exploratory efficacy endpoints included the percentage of patients with IBS‐M that were API weekly responders over 12 weeks; the change from baseline at week 12 in weekly average number of days with bowel urgency episodes (defined as the urgent need to rush to the restroom for a bowel movement), recurrent bowel movements (defined as >1 bowel movement in any 1‐hour period), and diarrhea (defined as Bristol type 6 or 7 stool) for all patients with IBS; and the change from baseline in average daily number of bowel movements in all patients with IBS.

#### 2.2.2 Safety

Measures of safety included incidence of adverse events (AEs), clinical laboratory assessments, vital signs, and physical examinations. AEs and serious AEs were collected from the time of informed consent until follow‐up was completed. Treatment‐emergent AEs (TEAEs) were defined as AEs starting or worsening after the first dose of double‐blind study treatment through 14 days after the last dose.

### 2.3 Statistical analyses

The primary study objective was to estimate the treatment effect of vibegron relative to placebo with respect to improvement in IBS‐related abdominal pain in patients with IBS‐D. There was no formal statistical hypothesis testing. Nominal *p* values from comparisons to placebo may be provided for descriptive purposes. Outcomes were assessed in the full analysis set (FAS), which included all randomly assigned patients with IBS‐D or IBS‐M (dependent on population [i.e., IBS‐D, IBS‐M, IBS overall]) who took ≥1 dose of double‐blind study treatment and had ≥1 evaluable weekly API score. Efficacy endpoints were analyzed using a Cochran–Mantel–Haenszel (CMH) risk differences estimate stratified by baseline abdominal pain strata (<6 vs. ≥6) per randomization stratification with weights proposed by Greenland and Robins.^19^ The estimated common risk difference and associated nominal *p* value and 2‐sided 90% confidence interval (CI) was determined. A mixed model for repeated measures (MMRM) with restricted maximum likelihood estimation was used to analyze changes from baseline at week 12 in efficacy outcomes. Covariates included in the MMRM were treatment, study visit, baseline score, abdominal pain strata by actual baseline, and interaction by study visit interaction for IBS‐D or IBS‐M. Safety outcomes were analyzed in the safety set, which included all patients with IBS‐D and with IBS‐M who received ≥1 dose of double‐blind study treatment, and descriptive statistics of observed values were reported for each treatment group.

## 3 RESULTS

### 3.1 Study participants

**FIGURE 1:**
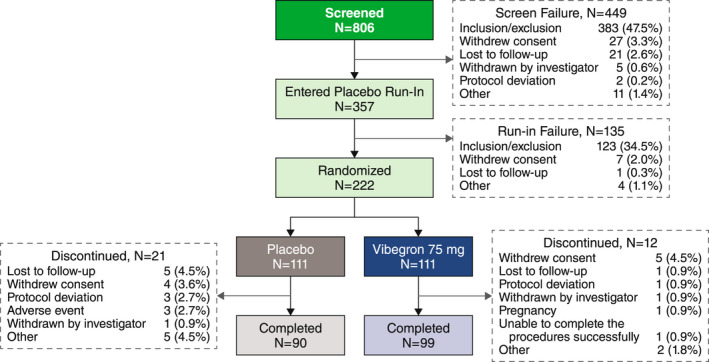
Patient disposition

The study was conducted from December 31, 2018, to October 6, 2020, at 26 sites in the United States. Among the 806 patients screened, 222 were randomly assigned to receive vibegron 75 mg (*n* = 111) or placebo (*n* = 111) (Figure 1). Of those randomized, 219 patients were included in the overall FAS: 129 (58.9%) in the IBS‐D group (vibegron, *n* = 63; placebo, *n* = 66) and 90 (41.1%) in the IBS‐M group (vibegron, *n* = 45; placebo, *n* = 45). Overall, 189 patients (85.1%) completed the 12‐week study, with comparable completion rates across treatment groups. Three patients receiving placebo discontinued study treatment owing to TEAEs, including elevated liver enzymes (aspartate aminotransferase, alanine aminotransferase) in 1 patient and worsening of IBS in 2 patients; no patients receiving vibegron discontinued study treatment owing to a TEAE.

Baseline characteristics were well balanced between treatment groups (Table 1). Overall, the mean age was 40.1 years, most (74%) patients were White, and 66.2% were of childbearing potential. At baseline, the API score and weekly mean of worst daily mean pain score were similar in women with IBS‐D or with IBS‐M across treatment groups. Overall, 29.2% of patients used ≥1 rescue medication (Table S1).

**TABLE 1:** Patient Baseline Demographics and Clinical Characteristics

Characteristic	IBS‐D Population		IBS‐M Population		Overall Population (FAS)^a^	
	Placebo (*n* = 63)	Vibegron (*n* = 66)	Placebo (*n* = 45)	Vibegron (*n* = 45)	Placebo (*n* = 108)	Vibegron (*n* = 111)
Mean (SD) age, years	40.6 (14.3)	41.2 (14.3)	38.2 (11.2)	39.5 (13.4)	39.6 (13.1)	40.5 (13.9)
Age subgroup, *n* (%)						
<40 years	33 (52.4)	35 (53.0)	29 (64.4)	26 (57.8)	62 (57.4)	61 (55.0)
≥40 to <65 years	28 (44.4)	24 (36.4)	16 (35.6)	17 (37.8)	44 (40.7)	41 (36.9)
≥65 years	2 (3.2)	7 (10.6)	0	2 (4.4)	2 (1.9)	9 (8.1)
Race, *n* (%)						
White	51 (81.0)	48 (72.7)	31 (68.9)	32 (71.1)	82 (75.9)	80 (72.1)
Black or African American	11 (17.5)	14 (21.2)	13 (28.9)	11 (24.4)	24 (22.2)	25 (22.5)
Other	1 (1.6)	3 (4.5)	0	0	1 (0.9)	3 (2.7)
American Indian or Alaska Native	0	0	1 (2.2)	1 (2.2)	1 (0.9)	1 (0.9)
Asian	0	1 (1.5)	0	0	0	1 (0.9)
Native Hawaiian or other Pacific Islander	0	0	0	1 (2.2)	0	1 (0.9)
Childbearing potential, *n* (%)	41 (65.1)	45 (68.2)	31 (68.9)	28 (62.2)	72 (66.7)	73 (65.8)
Baseline API score, *n* (%)						
<6	44 (69.8)	46 (69.7)	33 (73.3)	32 (71.1)	77 (71.3)	78 (70.3)
≥6	19 (30.2)	20 (30.3)	12 (26.7)	13 (28.9)	31 (28.7)	33 (29.7)
Weekly mean (SD) worst daily pain score	5.0 (1.7)	5.1 (1.5)	5.3 (1.7)	5.1 (1.7)	5.1 (1.7)	5.1 (1.6)
Preexisting hypertension, *n* (%)^b^	20 (31.7)	12 (18.2)	11 (24.4)	8 (17.8)	31 (28.7)	20 (18.0)

Abbreviations: API, abdominal pain intensity; FAS, full analysis set; IBS‐D, irritable bowel syndrome with predominantly diarrhea; IBS‐M, irritable bowel syndrome with predominantly mixed episodes of diarrhea and constipation.

^a^ All randomized patients with IBS‐D or with IBS‐M at study entry who took ≥1 dose of double‐blind study treatment and had ≥1 evaluable weekly API score.

^b^ Preexisting hypertension was based on medical history and/or baseline hypertension defined as baseline systolic blood pressure ≥140 mmHg and/or diastolic blood pressure ≥90 mmHg.

### 3.2 Efficacy

#### 3.2.1 Abdominal pain intensity weekly responders

At week 12, there was no significant difference (90% CI of CMH difference includes 0) in the percentage of women with IBS‐D experiencing ≥30% decrease in weekly average of “worst possible abdominal pain in the past 24 hours” with vibegron vs. placebo (40.9% vs. 42.9%, respectively; CMH difference [90% CI], −1.9 [−16.1 to 12.3]; nominal *p* = 0.8222) (Table 2). No significant differences (90% CIs of CMH difference include 0) between vibegron and placebo were observed among patients with IBS‐D who were considered API weekly responders with ≥40% (33.3% vs. 31.7%, respectively; CMH difference [90% CI], 1.6 [−11.7 to 14.9]; nominal *p* = 0.8434) or ≥50% (27.3% vs. 20.6%, respectively; CMH difference [90% CI], 6.7 [−5.5 to 18.8]; nominal *p* = 0.3691) reduction from baseline at week 12. Similarly, there were no significant differences (90% CIs of CMH difference include 0) in the percentage of women with IBS‐M who were API weekly responders with ≥30% (CMH difference [90% CI], 4.7 [−10.6 to 19.9]; nominal *p* = 0.6151), ≥40% (CMH difference [90% CI], 6.9 [−5.8 to 19.7]; nominal *p* = 0.3706), or ≥50% (CMH difference [90% CI], 4.7 [−6.5 to 16.3]; nominal *p* = 0.5005) reduction from baseline at week 12 with vibegron vs. placebo.

**TABLE 2:** API Weekly Responder Analysis of Patients With IBS Achieving ≥30%, ≥40%, and ≥50% Reduction in API Score at Week 12^a^

Outcome	IBS‐D Population^b^		IBS‐M Population^b^	
	Placebo (*n* = 63)	Vibegron (*n* = 66)	Placebo (*n* = 45)	Vibegron (*n* = 45)
≥30% reduction at week 12				
Responder, *n* (%)	27 (42.9)	27 (40.9)	11 (24.4)	13 (28.9)
CMH difference (90% CI)^c^	–	−1.9 (−16.1 to 12.3)	–	4.7 (−10.6 to 19.9)
Nominal *p* value^c^	–	0.8222	–	0.6151
≥40% reduction at week 12				
Responder, *n* (%)	20 (31.7)	22 (33.3)	6 (13.3)	9 (20.0)
CMH difference (90% CI)^c^	–	−1.6 (−11.7 to 14.9)	–	6.9 (−5.8 to 19.7)
Nominal *p* value^c^	–	0.8434	–	0.3706
≥50% reduction at week 12				
Responder, *n* (%)	13 (20.6)	18 (27.3)	5 (11.1)	7 (15.6)
CMH difference (90% CI)^c^	–	6.7 (−5.5 to 18.8)	–	4.7 (−6.8 to 16.3)
Nominal *p* value^c^	–	0.3691	–	0.5005

Abbreviations: API, abdominal pain intensity; CMH, Cochran–Mantel–Haenszel; IBS‐D, irritable bowel syndrome with predominantly diarrhea; IBS‐M, irritable bowel syndrome with mixed episodes of diarrhea and constipation.

^a^ Analyzed using the CMH risk difference estimate stratified by randomized baseline abdominal pain strata (<6 vs. ≥6), with weights proposed by Greenland and Robins.

^b^ All randomized patients with IBS‐D or with IBS‐M at study entry who took ≥1 dose of double‐blind study treatment and had ≥1 evaluable weekly API score.

^c^ Vibegron – placebo.

#### 3.2.2 Global improvement score responders

A greater percentage of patients with IBS‐D receiving vibegron were considered GIS responders at week 12 compared with placebo (42.4% vs. 33.3%, respectively), but the difference between the treatment groups was not significant (90% CI of CMH difference includes 0; CMH difference [90% CI], 9.1 [−4.8 to 22.9]; nominal *p* = 0.2821) (Table 3). There was no significant difference (90% CI of CMH difference includes 0) in the percentage of patients with IBS‐M treated with vibegron vs. placebo who were considered GIS responders at week 12 (CMH difference [90% CI], −0.1 [−16.7 to 16.4]; nominal *p* = 0.9892) (Table 3).

**TABLE 3:** Global Improvement Scale Responder Analysis at Week 12^a^

Parameter	IBS‐D Population^b^		IBS‐M Population^b^	
	Placebo (*n* = 63)	Vibegron (*n* = 66)	Placebo (*n* = 45)	Vibegron (*n* = 45)
Responder, *n* (%)	21 (33.3)	28 (42.4)	16 (35.6)	16 (35.6)
CMH difference (90% CI)^c^	–	9.1 (−4.8 to 22.9)	–	−0.1 (−16.7 to 16.4)
Nominal *p* value^c^	–	0.2821	–	0.9892

Abbreviations: CMH, Cochran–Mantel–Haenszel; IBS‐D, irritable bowel syndrome with predominantly diarrhea; IBS‐M, irritable bowel syndrome with mixed episodes of diarrhea and constipation.

^a^ Analyzed using the CMH risk difference estimate stratified by randomized baseline abdominal pain strata (<6 vs. ≥6), with weights proposed by Greenland and Robins.

^b^ All randomized patients with IBS‐D or with IBS‐M at study entry who took ≥1 dose of double‐blind study treatment and had ≥1 evaluable weekly API score.

^c^ Vibegron – placebo.

#### 3.2.3 Weekly average number of days with bowel urgency episodes, recurrent bowel movements, and diarrhea and average bowel frequency

Vibegron was associated with significant reductions vs. placebo from baseline at week 12 in least squares (LS) mean weekly average in number of days with bowel urgency episodes in the overall patient population with IBS (LS mean difference [90% CI], −0.8 [−1.4 to −0.1]; nominal *p* < 0.0434) (Table S2). No significant differences (90% CI of LS mean difference includes 0) were observed between vibegron and placebo in change from baseline at week 12 in weekly average number of days with recurrent bowel movements (LS mean difference [90% CI], −0.1 [−0.2 to 0.4]) and diarrhea (LS mean difference [90% CI], −0.2 [−0.8 to 0.4]). No significant difference (90% CI of LS mean difference includes 0) was observed between vibegron and placebo in change from baseline at week 12 in daily average number of bowel movements (LS mean difference [90% CI], 0.2 [−0.1 to 0.4]; Table S2).

### 3.3 Safety

The incidence of TEAEs was generally comparable between patients in the vibegron group and the placebo group (33.3% each) (Table 4). Serious TEAEs were reported in 1 patient (0.9%) in the placebo group (hyperkalemia) and in 2 patients (1.8%) in the vibegron group (COVID‐19 and ectopic pregnancy); however, no serious TEAE was considered by the investigator to be related to study treatment. The most commonly reported TEAEs (occurring in ≥2% of patients) in the vibegron group were bacteriuria, gastroenteritis, headache, and worsening of IBS symptoms (2.7% each) and in the placebo group were bacteriuria and upper respiratory tract infection (4.5% each), as well as headache, worsening of IBS symptoms, constipation, leukocyturia, and nasopharyngitis (2.7% each). AEs of worsening of IBS symptoms were reported at equal rates in the vibegron and placebo groups (2.7% each).

**TABLE 4:** Summary of AEs

AE, *n* (%)	Placebo (*n* = 111)	Vibegron (*n* = 111)
≥1 TEAE	37 (33.3)	37 (33.3)
≥1 serious TEAE	1 (0.9)	2 (1.8)
COVID‐19	0	1 (0.9)
Ectopic pregnancy	0	1 (0.9)
Hyperkalemia	1 (0.9)	0
TEAEs occurring in ≥2% of patients in any group by SOC		
Infections and infestations		
Bacteriuria	5 (4.5)	3 (2.7)
Gastroenteritis	0	3 (2.7)
Upper respiratory tract infection	5 (4.5)	1 (0.9)
Nasopharyngitis	3 (2.7)	0
Gastrointestinal disorders		
Worsening of IBS symptoms	3 (2.7)	3 (2.7)
Constipation	3 (2.7)	1 (0.9)
Nervous system disorders		
Headache	3 (2.7)	3 (2.7)
Renal and urinary disorders		
Leukocyturia	3 (2.7)	0

Abbreviations: AE, adverse event; IBS, irritable bowel syndrome; SOC, system organ class; TEAE, treatment‐emergent AE.

## 4 DISCUSSION

In this phase 2, prospective, randomized, placebo‐controlled, proof‐of‐concept trial, no significant or clinically relevant difference was observed with vibegron compared with placebo for the primary endpoint of the percentage of patients classified as API weekly responders (i.e., ≥30% improvement in abdominal pain associated with IBS at week 12 in women with IBS‐D). Similar results were observed among women with IBS‐D considered API weekly responders with ≥40% or ≥50% improvement from baseline at week 12 vs. placebo. Although a higher percentage of women with IBS‐D were considered GIS responders at week 12 with vibegron compared with placebo, the difference between treatment groups was not statistically significant. Furthermore, no clinically relevant differences were observed in the percentage of women with IBS‐M who were considered API weekly responders and GIS responders with vibegron compared with placebo. In the overall IBS population, vibegron was associated with significant improvement in the weekly average number of days with bowel urgency episodes compared with placebo. This is clinically relevant as many patients with IBS‐D symptoms rate urgency as one of their most bothersome symptoms.^20^ However, no significant differences in weekly average number of days with recurrent bowel movements or with diarrhea were observed between treatment groups.

Although a relevant treatment difference was not observed in the efficacy analysis in this proof‐of‐concept‐study, safety results showed that once‐daily vibegron 75 mg for 12 weeks was generally safe and well tolerated in women with IBS. Patients who received vibegron had generally similar rates of TEAEs as those who received placebo. Further, no clinically meaningful differences in overall rates of TEAEs, of serious TEAEs, or of AEs leading to treatment discontinuation were observed between vibegron and placebo treatment groups. Notably, the rates of worsening diarrhea and other gastrointestinal and IBS‐associated TEAEs were similar between treatment groups. Furthermore, few patients reported worsening of IBS symptoms with vibegron, with rates equal to placebo.

The similar rates of gastrointestinal and IBS‐associated TEAEs between treatment arms in this study are important findings because recent studies have noted significant overlap between IBS and OAB, the indication for which vibegron is currently approved. Indeed, a survey of 10,000 respondents showed that 33% of adults with OAB have comorbid IBS (any subtype) compared with 20% of adults without OAB.^21^ The prevalence of IBS in adults with severe symptoms of OAB (based on OAB symptom scores) increased to 39%.^21^ Similarly, a survey of >5000 adults reported that among respondents with OAB, 27% have comorbid IBS (any subtype) compared with 12.3% of respondents without OAB.^22^ Nonetheless, the consistent safety profile of vibegron is clinically important because treatment with once‐daily vibegron 75 mg showed efficacy, safety, and tolerability in adults with OAB in the 12‐week phase 3 EMPOWUR trial, with headache reported as the most frequently occurring TEAE with vibegron reported at a higher rate than placebo (4.0% vs. 2.4%, respectively).^15^ Vibegron was approved in the US in 2020 for the treatment of OAB.

The investigation of new therapies for the treatment of IBS is difficult owing to the heterogeneity of the underlying pathophysiology and clinical presentation of IBS. Pharmacologic therapies for IBS focus on targeting the predominant bowel habit (i.e., IBS‐D, IBS‐C)^5^; however, there is no validated treatment algorithm.^5^ Additionally, there are no approved pharmacologic therapies for IBS‐M, as studies often neglect to include this subset of patients with IBS.^6^ Therefore, there is limited high‐quality evidence supporting the efficacy of pharmacologic therapies for treatment of IBS.^6^ Furthermore, a previous report has suggested that a combination of treatments, rather than monotherapy, is more likely to be beneficial among patients with IBS‐D.^23^

Limitations of these analyses include that this was a phase 2 proof‐of‐concept study that was based on estimation methods and not statistical hypothesis testing. Additionally, a relatively high placebo response rate was observed; however, this is consistent with previous short‐term trials showing a high 30%–80% placebo response rate in patients with IBS.^24^ Although this study was performed during the COVID‐19 global pandemic, a high percentage of patients (85.1%) completed the 12‐week study.

## 5 CONCLUSION

In this phase 2a study of women with IBS‐D or with IBS‐M, treatment with once‐daily vibegron 75 mg was not associated with significant clinical improvement of the key symptoms of IBS, including abdominal pain. Vibegron was generally safe and well tolerated among the overall patient population. Notably, patients treated with vibegron did not experience any clinical changes associated with IBS symptoms or worsening of IBS symptoms (i.e., diarrhea) compared with placebo.

## AUTHOR CONTRIBUTIONS

**BE Lacy** participated in the conceptualization and design of the research study, curation and analysis of data, and in the review and editing of the manuscript. **J King** participated in the design and methodology of the research study, data analysis, and in the review and editing of the manuscript. **C Schaumburg** participated in the design of the research study, data curation and analysis, and in the review and editing of the manuscript. **C Haag‐Molkenteller** participated in the design, methodology, and supervision of the research study; data analysis; and in the review and editing of the manuscript. **D Shortino** participated in the design of the research study, data curation and analysis, and in the review and editing of the manuscript. **WD Chey** participated in the design and conceptualization of the research study, curation and analysis of data, and in the review and editing of the manuscript.

## FUNDING INFORMATION

This study and medical writing and editorial support for the preparation of this manuscript were funded by Urovant Sciences (Irvine, CA).

## CONFLICT OF INTEREST

**BE Lacy** is a consultant and/or on the scientific advisory boards for Allakos, Alpha Sigma, Arena Pharmaceuticals, Ironwood, Salix, Sanofi, and Viver and serves on the Rome Board of Directors. **J King**, **D Shortino, C Schaumburg**, and **C Haag‐Molkenteller** were employees of Urovant Sciences at the time the work was conducted. **WD Chey** is a consultant for Abbvie, Allakos, Alnylam, Arena, Bayer, Biomerica, Ironwood, Nestle, QOL Medical, Salix/Valeant, Takeda, Urovant Sciences, and Vibrant; has received grant and/or research study funding from Biomerica, Commonwealth Diagnostics International, QOL Medical, and Salix; has stock options in GI on Demand, Modify Health, and Ritter; serves on the Rome Board of Directors; and is a member of the Board of Trustees of the American College of Gastroenterology and Board of Directors of the International Foundation for Gastrointestinal Disorders.

## Supporting information


TableS1‐S2

## References

[1] Sperber AD , Bangdiwala SI , Drossman DA , et al. Worldwide prevalence and burden of functional gastrointestinal disorders, results of Rome foundation global study. Gastroenterology. 2021;160:99‐114. 32294476 10.1053/j.gastro.2020.04.014

[2] Longstreth GF , Thompson WG , Chey WD , Houghton LA , Mearin F , Spiller RC . Functional bowel disorders. Gastroenterology. 2006;130:1480‐1491. 16678561 10.1053/j.gastro.2005.11.061

[3] Chey WD , Kurlander J , Eswaran S . Irritable bowel syndrome: a clinical review. Jama. 2015;313:949‐958. 25734736 10.1001/jama.2015.0954

[4] Lacy BE , Mearin F , Chang L , et al. Bowel disorders. Gastroenterology. 2016;150:1393‐1407. 10.1053/j.gastro.2016.02.031 27144627

[5] Ford AC , Moayyedi P , Chey WD , et al. American College of Gastroenterology monograph on management of irritable bowel syndrome. Am J Gastroenterol. 2018;113:1‐18. 10.1038/s41395-018-0084-x 29950604

[6] Lacy BE , Pimentel M , Brenner DM , et al. ACG clinical guideline: management of irritable bowel syndrome. Am J Gastroenterol. 2021;116:17‐44. 33315591 10.14309/ajg.0000000000001036

[7] Palsson OS , Whitehead W , Tornblom H , Sperber AD , Simren M . Prevalence of Rome IV functional bowel disorders among adults in the United States, Canada, and the United Kingdom. Gastroenterology. 2020;158:1262‐1273. 31917991 10.1053/j.gastro.2019.12.021

[8] De Ponti F , Gibelli G , Croci T , Arcidiaco M , Crema F , Manara L . Functional evidence of atypical beta 3‐adrenoceptors in the human colon using the beta 3‐selective adrenoceptor antagonist, SR 59230A. Br J Pharmacol. 1996;117:1374‐1376. 8730727 10.1111/j.1476-5381.1996.tb15294.x PMC1909449

[9] Krief S , Lonnqvist F , Raimbault S , et al. Tissue distribution of beta 3‐adrenergic receptor mRNA in man. J Clin Invest. 1993;91:344‐349. 8380813 10.1172/JCI116191 PMC330032

[10] Cellek S , Thangiah R , Bassil AK , et al. Demonstration of functional neuronal beta3‐adrenoceptors within the enteric nervous system. Gastroenterology. 2007;133:175‐183. 17631141 10.1053/j.gastro.2007.05.009

[11] Grudell AB , Camilleri M , Jensen KL , et al. Dose‐response effect of a beta3‐adrenergic receptor agonist, solabegron, on gastrointestinal transit, bowel function, and somatostatin levels in health. Am J Physiol Gastrointest Liver Physiol. 2008;294:G1114‐G1119. 18372395 10.1152/ajpgi.00051.2008

[12] Kelleher DL , Hicks KJ , Cox DS , Williamson RR , Alpers DH , Dukes GE . Randomized, double‐blind, placebo (PLA)‐controlled, crossover study to evaluate efficacy and safety of the beta 3‐adrenergic receptor agonist solabegron (SOL) in patients with irritable bowel syndrome (IBS). Neurogastroenterol Motil. 2008;20:131‐132.

[13] Brucker BM , McHale K , King J , Mudd PN. Selectivity and maximum response of vibegron and mirabegron for β_3_‐adrenergic receptors. Society of Urodynamics, Female Pelvic Medicine and Urogenital Reconstruction 2021 Winter Meeting; 2021 February 25‐27; Virtual Congress

[14] Urovant Sciences, Inc. GEMTESA^®^ (vibegron) . Full Prescribing Information: Urovant Sciences, Inc: 2020.

[15] Staskin D , Frankel J , Varano S , Shortino D , Jankowich R , Mudd PN Jr . International phase III, randomized, double‐blind, placebo and active controlled study to evaluate the safety and efficacy of vibegron in patients with symptoms of overactive bladder: EMPOWUR. J Urol. 2020;204:316‐324. 32068484 10.1097/JU.0000000000000807

[16] Staskin D , Frankel J , Varano S , Shortino D , Jankowich R , Mudd PN Jr . Once‐daily vibegron 75 mg for overactive bladder: long‐term safety and efficacy from a double‐blind extension study of the international phase 3 trial (EMPOWUR). J Urol. 2021;205:1421‐1429. 33356445 10.1097/JU.0000000000001574

[17] Gordon S , Ameen V , Bagby B , Shahan B , Jhingran P , Carter E . Validation of irritable bowel syndrome global improvement scale: an integrated symptom end point for assessing treatment efficacy. Dig Dis Sci. 2003;48:1317‐1323. 12870789 10.1023/a:1024159226274

[18] Lembo T , Wright RA , Bagby B , et al. Alosetron controls bowel urgency and provides global symptom improvement in women with diarrhea‐predominant irritable bowel syndrome. Am J Gastroenterol. 2001;96:2662‐2670. 11569692 10.1111/j.1572-0241.2001.04128.x

[19] Greenland S , Robins JM . Estimation of a common effect parameter from sparse follow‐up data. Biometrics. 1985;41:55‐68. 4005387

[20] Mangel AW , Wang J , Sherrill B , Gnanasakthy A , Ervin C , Fehnel SE . Urgency as an endpoint in irritable bowel syndrome. Gastroenterology Res. 2011;4:9‐12. 27957006 10.4021/gr283e PMC5139794

[21] Matsumoto S , Hashizume K , Wada N , et al. Relationship between overactive bladder and irritable bowel syndrome: a large‐scale internet survey in Japan using the overactive bladder symptom score and Rome III criteria. BJU Int. 2013;111:647‐652. 23106867 10.1111/j.1464-410X.2012.11591.x PMC3654175

[22] Matsuzaki J , Suzuki H , Fukushima Y , et al. High frequency of overlap between functional dyspepsia and overactive bladder. Neurogastroenterol Motil. 2012;24:821‐827. 22616664 10.1111/j.1365-2982.2012.01939.x

[23] Rusu F , Dumitrascu DL . Four years follow‐up of patients with irritable bowel syndrome. Rom J Intern Med. 2015;53:63‐72. 26076563 10.1515/rjim-2015-0009

[24] Talley NJ . Pharmacologic therapy for the irritable bowel syndrome. Am J Gastroenterol. 2003;98:750‐758. 12738451 10.1111/j.1572-0241.2003.07306.x

