# Supplementary material for: Efficacy and safety of vibegron for the treatment of irritable bowel syndrome in women: Results of a randomized, double‐blind, placebo‐controlled phase 2 trial
Source: Neurogastroenterol Motil. 2022 Aug 16;34(12):e14448. doi: 10.1111/nmo.14448 (PMC10078113; doi:10.1111/nmo.14448)
Supplement: Supplementary file 1 — TableS1‐S2 [file NMO-34-0-s001.docx]

Supplementary Table 1. Proportions of Patients With Concomitant Rescue Medication Use By Medication Classification

| **Characteristic** | **Placebo**  **(n=108)** | **Vibegron**  **(n=111)** |
| --- | --- | --- |
| Patients with ≥1 concomitant rescue medication | 26 (24.1) | 38 (34.2) |
| Antidiarrheals, intestinal anti-inflammatory/ anti-infective agents* | 12 (11.1) | 18 (16.2) |
| Pain | 5 (4.6) | 9 (8.1) |
| Stool | 7 (6.5) | 11 (9.9) |
| Pain and stool | 5 (4.6) | 11 (9.9) |
| Anti-inflammatory and antirheumatic agents^†^ | 6 (5.6) | 19 (17.1) |
| Pain | 5 (4.6) | 17 (15.3) |
| Stool | 0 | 3 (2.7) |
| Pain and stool | 3 (2.8) | 7 (6.3) |
| Analgesics^‡^ | 7 (6.5) | 6 (5.4) |
| Pain | 7 (6.5) | 6 (5.4) |
| Stool | 0 | 0 |
| Pain and stool | 0 | 1 (0.9) |
| Drugs for constipation^§^ | 4 (3.7) | 4 (3.6) |
| Pain | 2 (1.9) | 0 |
| Stool | 4 (3.7) | 4 (3.6) |
| Pain and stool | 2 (1.9) | 0 |
| Drugs for functional gastrointestinal disorders^¶^ | 2 (1.9) | 0 |
| Pain | 0 | 0 |
| Stool | 0 | 0 |
| Pain and stool | 2 (1.9) | 0 |

*Loperamide, loperamide hydrochloride, atropine sulfate, diphenoxylate hydrochloride, bismuth subsalicylate.

^†^Ibuprofen.

^‡^Acetaminophen.

^§^Macrogol 3350, macrogol, bisacodyl, liquid paraffin.

^¶^Artemisia dracunculus, carum carvi seed, coriandrum sativum seed, foeniculum vulgare essential oil, mentha X piperita, pimpinella anisum seed, zingiber officinale, dicycloverine.

Supplementary Table 2. Change from Baseline in Week 12 in Weekly Average Number of Days With Bowel Urgency Episodes, Recurrent Bowel Movements, and Diarrhea and in Average Number of Bowel Movements in Patients With IBS*

| **Characteristic** | **Placebo**  **(n=108)** | **Vibegron**  **(n=111)** |
| --- | --- | --- |
| Bowel urgency episodes | | |
| n | 65 | 65 |
| LS mean (SE) | ‒1.28 (0.277) | ‒2.04 (0.270) |
| LSMD (90% CI)^†^ | ‒ | ‒0.76 (–1.38 to –0.14) |
| Nominal *P* value^†^ | ‒ | 0.0434 |
| Recurrent bowel movements | | |
| n | 65 | 65 |
| LS mean (SE) | ‒0.37 (0.138) | ‒0.28 (0.135) |
| LSMD (90% CI)^†^ | ‒ | 0.09 (–0.22 to –0.40) |
| Nominal *P* value^†^ | ‒ | 0.6263 |
| Diarrhea^‡^ | | |
| n | 65 | 65 |
| LS mean (SE) | ‒1.21 (0.277) | ‒1.43 (0.266) |
| LSMD (90% CI)^†^ | ‒ | ‒0.22 (–0.84 to –0.39) |
| Nominal *P* value^†^ | ‒ | 0.5496 |
| Bowel frequency |  |  |
| n | 65 | 65 |
| LS mean (SE) | ‒0.33 (0.100) | ‒0.17 (0.096) |
| LSMD (90% CI)^†^ | ‒ | 0.16 (–0.07 to 0.38) |
| Nominal *P* value^†^ | ‒ | 0.2445 |

IBS, irritable bowel syndrome; LS, least squares; LSMD, LS mean difference.

*Analyzed using a mixed model for repeated measures with covariates for study visit, baseline value, abdominal pain strata by actual baseline, IBS subtype, and treatment by study visit interaction.

^†^Vibegron ‒ placebo.

^‡^Defined as Bristol Type 6 or 7.
